# Risk factors for SARS-CoV-2 infection and severe COVID-19 in unvaccinated solid organ transplant recipients

**DOI:** 10.1038/s41598-024-78119-6

**Published:** 2024-11-02

**Authors:** Casper Vrij, Kris Bogaerts, Pieter Vermeersch, Katrien Lagrou, Geert Molenberghs, Filip Rega, Laurens J. Ceulemans, Dirk Van Raemdonck, Ina Jochmans, Diethard Monbaliu, Jacques Pirenne, Geert Robaeys, Bart De Moor, Tim Vanuytsel, Pieter Gillard, Hélène Schoemans, Johan Van Cleemput, Dirk Kuypers, Robin Vos, Frederik Nevens, Jef Verbeek

**Affiliations:** 1 Department of Gastroenterology and Hepatology, University Hospitals Leuven, Leuven, Belgium; 2 Laboratory of Hepatology, CHROMETA, KU Leuven, Leuven, Belgium; 3 Department of Public Health and Primary Care, I-BioStat, KU Leuven and Hasselt University, Leuven, Belgium; 4 Department of Laboratory Medicine, University Hospitals Leuven, Leuven, Belgium; 5 Department of Microbiology, Immunology and Transplantation, KU Leuven, Leuven, Belgium; 6 Department of Cardiac Surgery, University Hospitals Leuven, Leuven, Belgium; 7 Laboratory of Cardiovascular Sciences, KU Leuven, Leuven, Belgium; 8 Department of Thoracic Surgery, University Hospitals Leuven, Leuven, Belgium; 9 Laboratory of Respiratory Diseases and Thoracic Surgery (BREATHE), CHROMETA, KU Leuven, Leuven, Belgium; 10 Department of Abdominal Transplant Surgery, University Hospitals Leuven, Leuven, Belgium; 11 Laboratory of Microbiology, Immunology and Transplantation, KU Leuven, Leuven, Belgium; 12 Department of Gastroenterology and Hepatology, Ziekenhuis Oost-Limburg Genk, Genk, Belgium; 13 Department of Nephrology, Jessa Hospital Hasselt, Hasselt, Belgium; 14 Department of Endocrinology, University Hospitals Leuven, Leuven, Belgium; 15 Laboratory of Clinical and Experimental Endocrinology, CHROMETA, KU Leuven, Leuven, Belgium; 16 Department of Hematology, University Hospitals Leuven, Leuven, Belgium; 17 Department of Public Health and Primary Care, ACCENT VV, KU Leuven, Leuven, Belgium; 18 Department of Cardiology, University Hospitals Leuven, Leuven, Belgium; 19 Department of Nephrology and Renal Transplantation, University Hospitals Leuven, Leuven, Belgium; 20 Department of Respiratory Diseases, University Hospitals Leuven, Leuven, Belgium

**Keywords:** Risk factors, SARS-CoV-2

## Abstract

The role of immunosuppressive therapy on SARS-CoV-2 infection risk and COVID-19 severity remains unclear in unvaccinated solid organ transplant recipients. We included 1957 organ transplant recipients between July 2020 and April 2021 to analyze whether baseline immunosuppressive therapy and other risk factors are associated with SARS-CoV-2 infection and severe COVID-19. In total, 247 (12.6%) had SARS-CoV-2 (defined as positive nasopharyngeal swab and/or positive antibody titer). Of these, 57 (23.1%) had severe COVID-19, defined as oxygen supplementation, intensive care unit admission or death. Multivariable analysis identified diabetes (hazard ratio (HR) 1.39 (95% confidence interval (CI) 1.05–1.83)), chronic lung disease (HR 1.71 (95% CI 1.13–2.60)) and contact with a COVID-19 positive individual (HR 3.61 (95% CI 2.61–4.99) as independent risk factors for SARS-CoV-2 infection. There was no association between immunosuppressive therapy and infection risk. Severe COVID-19 was multivariably associated with hypertension (OR 5.45 (95% CI 1.66–17.84)), chronic kidney disease (OR 3.55 (95% CI 1.75–7.19)), corticosteroid use (OR 2.93 (95% CI 1.03–2.55)) and having a COVID-19 positive housemate (OR 6.77 (95% CI 2.65–17.28)). In conclusion, baseline corticosteroid use, but no other immunosuppressive agent, is independently associated with severe COVID-19 in unvaccinated SOT recipients after correction for hypertension, chronic kidney disease, housemates affected by COVID-19 and transplant type.

**Supplementary Information:**

The online version contains supplementary material available at 10.1038/s41598-024-78119-6.

## Introduction

Solid organ transplant recipients (SOTs) are a risk population for severe coronavirus disease 19 (COVID-19)^1–3^ and are prioritized for vaccination^4^. Initial cohort studies during the early waves reported high rates of hospitalization (ranging from 78 to 89%)^5,6^ and mortality (ranging from 18 to 30%)^7^ in unvaccinated SOTs after infection with severe acute respiratory syndrome coronavirus 2 (SARS-CoV-2)^7–9^. Importantly, many of the initial studies regarding COVID-19 in unvaccinated SOT recipients were based on registries, which are subject to selection and reporting bias^7^, and thus primarily included SOTs with severe disease. But, similarly to immunocompetent individuals, a substantial proportion of SOTs undergo mild or even asymptomatic SARS-CoV-2 infections^10,11^. Furthermore, SARS-CoV-2 infection in these studies was often determined using RT-PCR-testing, which has a sensitivity that rapidly declines after the onset of symptoms^12^.

After the first waves of COVID-19 and the successful initiation of vaccination programs, it was quickly discovered that seroconversion after SARS-CoV-2 vaccination is diminished in SOTs^13^. We and several other studies identified older age, chronic kidney disease (CKD), higher daily dose of mycophenolate mofetil (MMF) and corticosteroids as risk factors for vaccine non-response^14–16^. Similarly, older age, MMF and corticosteroids were also risk factors for severe breakthrough infection in vaccinated SOTs^15,17–19^. Whether immunosuppressive agents are risk factors for severe disease due to their negative impact on vaccine response or via more direct pro-viral immunological effects remains elusive.

The fatal outcome of COVID-19 is in part modulated by hyperinflammation, which might be inhibited by immunosuppressive agents^20^. Calcineurin inhibitors (CNI) have been shown to possess in vitro inhibitory effects on SARS-CoV-2^21^. However, inconsistent data have been published on the role of different types of baseline immunosuppressive therapy as an independent risk factor for severe COVID-19 in unvaccinated transplant recipients^22^. Tacrolimus has been identified as a protective factor for severe COVID-19 in unvaccinated liver transplant recipients^23^, but not in other studies^22^. One study identified MMF as an independent risk factor for severe COVID-19 in unvaccinated liver transplant recipients^24^, but this association was not confirmed by others^22,25,26^.

Despite the widespread advocation for vaccination, initial expressed hesitancy for SARS-CoV-2 vaccination ranged from 34 − 70% in SOTs^27,28^, with 11.6% reporting to refuse vaccination in the latter study^28^. The willingness for SOTs to continue SARS-CoV-2 vaccination in the future is unclear, however, annual vaccination rates for influenza have been reported to be as low as 25% in SOTs^29^. Furthermore, adherence to preventive measures decreased after SARS-CoV-2 vaccination in a study of kidney transplant recipients^30^. Given the possible vaccine hesitancy and lower vaccination rates in SOTs in the future, it is valuable to have adequate insight into the risk factors for acquiring SARS-CoV-2 and severe COVID-19 in unvaccinated SOTs. In this study, we analyzed several risk factors with emphasis on immunosuppressive baseline therapy for acquiring SARS-CoV-2 infection and for severe COVID-19, in a large cohort of all types of SOTs in the pre-vaccination era.

## Results

### Patient characteristics

A total of 1957 SOT recipients were included, consisting of 566 (28.9%) lung, 566 (28.9%) kidney, 326 (16.6%) liver, 318 (16.2%) heart and 178 (9.1%) combined-organ transplant recipients. The latter group also contained 2 intestinal and 1 single pancreas transplant recipient. Baseline characteristics, including baseline immunosuppressive therapy, are presented in Table 1. The mean age was 58 years (± SD 14), with a male predominance of 59.1%. The most common co-morbidities were hypertension (81.3%), chronic kidney disease (51.0%) and diabetes (28.8%). Most patients lived with one or more housemates (87.0%) and a vast majority reported to always or almost always comply with the government issued COVID-19 rules (98.6%).

**Table 1:** Baseline patient characteristics of included SOT recipients.

	Transplant						
	Only kidney	Only heart	Only liver	Only lung	Multi-organ	Total*	
Male (%)	338/566 (59.7%)	231/318 (72.6%)	200/326 (61.3%)	291/566 (51.4%)	95/178 (53.4%)	1157/1957 (59.1%)	
Age (years)	58 (13)	58 (16)	61 (14)	58 (13)	56 (13)	58 (14)	
BMI (kg/m^2^)	25 (5)	26 (5)	27 (5)	25 (5)	24 (4)	25 (5)	
Time between last transplant and baseline visit (years)	10.2 (8.9)	11.3 (7.6)	7.8 (7.1)	6.5 (4.9)	8.0 (7.0)	8.7 (7.3)	
Co-morbidities							
Obesity (BMI ≥ 30 kg/m^2^)	82/526 (15.6%)	50/312 (16.0%)	73/314 (23.2%)	69/545 (12.7%)	14/170 (11.5%)	288/1870 (15.4%)	
Hypertension	499/566 (88.2%)	252/318 (79.2%)	216/326 (66.3%)	495/566 (87.5%)	129/178 (72.5%)	1592/1957 (81.3%)	
Diabetes	160/566 (28.3%)	71/318 (22.3%)	108/326 (33.1%)	181/566 (32.0%)	43/178 (24.2%)	563/1957 (28.8%)	
Chronic kidney disease	368/ 566 (65.0%)	149/318 (46.9%)	106/325 (32.6%)	287/566 (50.7%)	85/178 (47.8%)	997/1956 (51.0%)	
Chronic heart disease	87/566 (15.4%)	70/318 (22.0%)	42/326 (12.9%)	72/566 (12.7%)	30/178 (16.9%)	301/1957 (15.4%)	
Chronic lung disease	20/566 (3.5%)	16/318 (5.0%)	13/326 (4.0%)	67/566 (11.8%)	14/178 (7.9%)	131/1957 (6.7%)	
Chronic liver disease	4/566 (0.7%)	3/318 (0.9%)	6/326 (1.8%)	16/566 (2.8%)	1/178 (0.6%)	30/1957 (1.5%)	
Chronic neurological disease	29/566 (5.1%)	48/318 (15.1%)	16/326 (4.9%)	26/566 (4.6%)	7/178 (3.9%)	126/1957 (6.4%)	
Known HIV	2/566 (0.4%)	0/318 (0.0%)	1/326 (0.3%)	1/566 (0.2%)	0/178 (0.0%)	4/1957 (0.2%)	
Current or past malignancy (solid organ or hematological; excl. skin tumors)	39/566 (6.9%)	18/ 318 (5.7%)	46/326 (14.1%)	20/566 (3.5%)	12/178 (6.7%)	135/1957 (6.9%)	
Chemo-/immunotherapy against malignancy in 2020	5/564 (0.9%)	1/318 (0.3%)	0/326 (0.0%)	1/566 (0.2%)	1/177 (0.6%)	8/1954 (0.4%)	
Immunosuppression							
Tacrolimus	461/566 (81.4%)	267/318 (84.0%)	292/326 (89.6%)	509/566 (89.9%)	164/178 (92.1%)	1695/1957 (86.6%)	
Tacrolimus through level for all patients—Median (Q1 ; Q3)	7.1 (6.1; 8.4)	7.1 (5.9; 8.2)	4.7 (3.4; 6.1)	6.6 (5.5; 8.1)	7.0 (5.7; 8.3)	6.6 (5.3; 8.0)	
Cyclosporine	79/566 (14.0%)	44/318 (13.8%)	24/326 (7.4%)	52/566 (9.2%)	10/178 (5.6%)	209/1957 (10.7%)	
Cyclosporin through level for all patients—Median (Q1 ; Q3)	93.0 (72.7; 117.0)	107.5 (95.2; 123.0)	59.2 (45.4; 90.2)	167.5 (119.5; 234.0)	122.0 (54.4; 145.0)	107.0 (80.2; 139.0)	
Mycophenolate	410/566 (72.4%)	247/318 (77.7%)	138/ 326 (42.3%)	309/566 (54.6%)	119/178 (66.9%)	1223/1957 (62.5%)	
Mycophenolate daily dosage (mg)—Median (Q1 ; Q3)	1000 (500; 1000)	1000 (750; 2000)	500 (500; 500)	1000 (750; 1500)	1000 (500; 1000)	1000 (500; 1000)	
Azathioprine	37/566 (6.5%)	13/318 (4.1%)	11/326 (3.4%)	146/566 (25.8%)	14/178 (7.9%)	221/1957 (11.3%)	
Azathioprine daily dosage (mg)—Median (Q1 ; Q3)	75.0 (50.0; 100.0)	50.0 (25.0; 50.0)	50.0 (50.0; 75.0)	25.0 (25.0; 50.0)	50.0 (25.0; 50.0)	50.0 (25.0; 50.0)	
Corticosteroids (methylprednison)	330/566 (58.3%)	59/318 (18.6%)	38/326 (11.7%)	562/566 (99.3%)	108/178 (60.7%)	1099/1957 (56.2%)	
Corticosteroids daily dosage (mg)—Median (Q1 ; Q3)	4.0 (4.0; 4.0)	4.0 (2.0; 4.0)	4.0 (4.0; 12.0)	4.0 (4.0; 4.0)	4.0 (4.0; 4.0)	4.0 (4.0; 4.0)	
Everolimus	6/566 (1.1%)	22/318 (6.9%)	50/326 (15.3%)	36/566 (6.4%)	6/178 (3.4%)	122/1957 (6.2%)	
Everolimus trough level (µg/L)—Median (Q1 ; Q3)	5.4 (5.4; 6.6)	5.2 (4.0; 6.2)	3.3 (2.5; 3.7)	4.2 (3.4; 5.0)	3.3 (2.6; 5.1)	3.7 (2.9; 4.9)	
Sirolimus	3/566 (0.5%)	0/318 (0.0%)	0/326 (0.0%)	0/ 566 (0.0%)	1/178 (0.6%)	4/1957 (0.2%)	
Sirolimus peak-off level (µg/L)—Median (Q1 ; Q3)	4.3 (3.6; 7.4)	–	–	–	5.8 (5.8; 5.8)	5.0 (3.9; 6.6)	
Social risk factors							
Number of housemates (excl. patient)							
0	56/524 (10.7%)	48/308 (15.6%)	34/303 (11.2%)	77/523 (14.7%)	22/162 (13.6%)	237/1823 (13.0%)	
1	259/524 (49.4%)	161/308 (52.3%)	161/303 (53.1%)	264/523 (50.5%)	76/162 (46.9%)	924/1823 (50.7%)	
2	115/524 (21.9%)	44/308 (14.3%)	51/303 (16.8%)	100/523 (19.1%)	30/162 (18.5%)	340/1823 (18.7%)	
> 2	94/524 (17.9%)	55/308 (17.8%)	57/303 (18.8%)	82/523 (16.2%)	34/162 (21.0%)	322/1823 (17.7%)	
COVID-19 suspicion among housemates							
Yes	23/505 (4.6%)	12/302 (4.0%)	16/300 (5.3%)	12/509 (2.4%)	5/156 (3.2%)	68/1775 (3.8%)	
No	426/505 (84.4%)	242/302 (80.1%)	250/300 (83.3%)	420/509 82.5%)	129/156 (82.7%)	1470/1775 (82.8%)	
Not applicable	56/505 (11.1%)	48/302 (15.9%)	34/300 (11.3%)	77/509 (15.1%)	22/156 (14.1%)	237/1775 (13.4%)	
Compliance with COVID-19 rules							
Always	377/546 (69.0%)	213/303 (70.3%)	275/318 (86.5%)	373/544 (68.6%)	121/167 (72.5%)	1361/1881 (72.4%)	
Almost always	161/546 (29.5%)	81/303 (26.7%)	41/318 (12.9%)	163/544 (30.0%)	45/167 (26.9%)	492/1881 (26.2%)	
Never	1/546 (0.2%)	1/303 (0.3%)	2/318 (0.6%)	2/544 (0.4%)	0/167 (0.0%)	6/1881 (0.3%)	
Compliance of housemates with COVID-19 rules							
Always	264/511 (51.7%)	151/306 (49.3%)	204/300 (68.0%)	235/506 (46.4%)	84/157 (53.5%)	939/1782 (52.7%)	
Almost always	174/511 (34.1%)	94/306 (30.7%)	55/300 (18.3%)	176/506 (34.8%)	45/157 (28.7%)	545/1782 (30.6%)	
Never	6/511 (1.2%)	0/306 (0.0%)	1/300 (0.3%)	3/506 (0.6%)	1/157 (0.6%)	11/1782 (0.6%)	
Not applicable	56/511 (11.0%)	48/306 (15.7%)	34/300 (11.3%)	77/506 (15.2%)	22/157 (14.0%)	237/1782 (13.3%)	
Patient bought food during complete lock-down							
Always	102/548 (18.6%)	44/308 (14.3%)	101/314 (32.2%)	59/549 (10.7%)	30/170 (17.6%)	337/1892 (17.8%)	
Almost always	44/548 (8.0%)	19/308 (6.2%)	25/314 (8.0%)	35/549 (6.4%)	10/170 (5.9%)	134/1892 (7.1%)	
Never	176/548 (32.1%)	168/308 (54.5%)	126/314 (40.1%)	249/549 (45.4%)	78/170 (45.9%)	798/1892 (42.2%)	
Patient wears mouth mask if social distance of 1.5 m cannot be kept							
Always	482/549 (87.8%)	272/307 (88.6%)	275/317 (86.8%)	483/549 (88.0%)	145/169 (85.8%)	1660/1894 (87.6%)	
Almost always	55/549 (10.0%)	27/307 (8.8%)	22/317 (6.9%)	47/549 (8.6%)	15/169 (8.9%)	166/1894 (8.8%)	
Never	1/549 (0.2%)	0/307 (0.0%)	4/317 (1.3%)	2/549 (0.4%)	2/169 (1.2%)	9/1894 (0.5%)	
Spent at least 1 night abroad since February 4th 2020	48/ 346 (13.9%)	44/ 212 (20.8%)	32/ 190 (16.8%)	40/ 299 (13.4%)	12/ 101 (11.9%)	178/1151 (15.5%)	

*The total column also includes 1 single-pancreas and 2-single intestine transplant recipients.

### Risk factors for SARS-CoV-2 infection in SOT recipients

From the 1957 included SOT recipients, 247 (12.6%) had a SARS-CoV-2 infection. SARS-CoV-2 infection was determined on the basis of a positive RT-PCR in 48 recipients, positive SARS-CoV-2 IgG antibody titer in 93 recipients, and both positive SARS-CoV-2 IgG antibodies and RT-PCR in 106 recipients. Among those with a SARS-CoV-2 infection, 178 (62.1%) patients had an infection before the moment of study inclusion. 69 (27.9%) were infected during follow-up. Median follow-up was 258 days (8.5 months), ranging from 38 to 451 days. 62 of 93 patients had only positive SARS-CoV-2 IgG anti-S in case of a positive antibody titre, while 18 patients had only IgG anti-N and 13 patients had both.

Using a Cox proportional hazard model, we found that SOT recipients with an infection more often had diabetes (35.4% vs. 27.8%, HR 1.40 (95% CI 1.07–1.82), *p* = 0.013), chronic lung diseases (11.4% vs. 6.0%, HR 1.96 (95% CI I1.32–2.92), *p* < 0.001) and had a lower daily MMF dose (median 566.7 mg vs. 651.2 mg; *p* = 0.002) compared to those without infection (Table 2). Additionally, recipients with an infection more frequently had a COVID-19 suspicion among housemates (HR 3.30 (95% CI 2.28–4.78) *p* < 0.001) and more frequently had been in close contact with an individual with confirmed COVID-19 (HR 3.71 (95% CI 2.72–5.07), *p* < 0.001). Chronic kidney disease was not a signficiant risk factor in the kidney transplant group (HR 0.67 (95% CI 0.39–1.14), *p* = 0.138), nor in the other transplant recipients (HR 0.98 (95% CI 0.74–1.31), *p* = 0.904). Results from univariable analysis can be found in supplementary Table 1.

**Table 2:** Characteristics of SOT recipients with versus without SARS-CoV-2 infection.

	No infection	Infection	*P*-value
Number of patients	1710	247	
Type of transplant			
Only kidney	507 (29.68%)	59 (23.87%)	0.447
Only heart	279 (16.31%)	39 (15.8%)	
Only liver	258 (15.08%)	68 (27.57%)	
Only lung	506 (29.59%)	60 (24.29%)	
Multi-organ*	160 (9.33%)	21 (8.66%)	
Male	1011 (59.10%)	146 (59.27%)	0.781
Age (years)	58.2 (13.48)	57.8 (15.36)	**0.041**
BMI ≥ 30	260 (15.21%)	45 (18.06%)	0.754
Medically treated hypertension	1401 (81.95%)	191 (77.17%)	**0.038**
Medically treated diabetes	476 (27.81%)	87 (35.38%)	**0.013**
Chronic kidney disease	887 (51.86%)	110 (44.62%)	0.194
Chronic heart disease	253 (14.80%)	48 (19.43%)	0.347
Chronic lung disease	103 (6.02%)	28 (11.38%)	**< 0.001**
Chronic liver disease	24 (1.40%)	6 (2.43%)	0.107
Chronic neurological disease	108 (6.30%)	18 (7.37%)	0.556
Known HIV	4 (0.23%)	0 (0.00%)	NA
Current or past malignancy (solid organ or hematological; excl. skin tumors)	122 (7.12%)	13 (5.34%)	0.230
Chemo-/immunotherapy against malignancy in 2020	9 (0.50%)	0 (0.00%)	0.972
Tacrolimus	1483 (86.75%)	216 (87.29%)	0.696
Tacrolimus off-peak level (µg/L)	6.0 (3.23)	5.7 (3.33)	0.142
Cyclosporine	186 (10.85%)	25 (10.28%)	0.380
Cyclosporine off-peak level (µg/L)	13.0 (41.87)	11.6 (40.82)	0.387
Mycophenolate	1106 (64.69%)	140 (56.88%)	0.055
Mycophenolate daily dose (mg)	651.2 (636.95)	566.7 (700.07)	**0.002**
Azathioprine	199 (11.62%)	25 (10.24%)	0.727
Azathioprine daily dose (mg)	75 (4.36%)	8 (3.04%)	0.770
Corticosteroids (methylprednisone)	997 (58.30%)	123 (49.64%)	0.543
Corticosteroids daily dose (4 mg)	699 (40.85%)	85 (34.53%)	0.829
Everolimus	113 (6.60%)	16 (6.56%)	0.724
Everolimus peak-off level (µg/L)	0.3 (1.09)	0.3 (1.07)	0.572
Number of housemates			
0	225 (13.14%)	32 (13.04%)	0.953
1	863 (50.48%)	125 (50.57%)	
> 1	622 (36.38%)	90 (36.40%)	
Covid suspicion among housemates	50 (2.94%)	44 (17.81%)	**< 0.001**
Been within 1.5 m of confirmed Covid patient	85 (4.98%)	65 (26.44%)	**< 0.001**
Always compliant with COVID rules	1242 (72.63%)	167 (67.53%)	0.551
Housemates (+ 16y) were always compliant with COVID rules	905 (52.94%)	127 (51.46%)	0.929
Patient never bought food during lock-down	718 (41.99%)	114 (46.23%)	0.057
Patient always wears mouth mask if social distance of 1.5 m cannot be kept	1528 (89.33%)	211 (85.55%)	0.181
Spent at least 1 night abroad since February 4th 2020	261 (15.25%)	44 (17.94%)	0.147

SARS-CoV-2 infection was defined as positive SARS-CoV-2 PCR and/or positive SARS-CoV-2 IgG antibody titre.

P-values are obtained from Cox PH model with missing data accounted for by performing 10 multiple imputations.

*The multi-organ transplant group also contains 1 single pancreas and 2 only intestine transplant recipients.

Significant values are given in bold.

Multivariable analysis (using a cox proportional hazard model) identified diabetes (HR 1.39 (95% CI 1.05–1.83), *p* = 0.021), chronic lung disease (HR 1.71 (95% CI 1.13–2.60), *p* = 0.012) and close contact with a confirmed COVID-19 individual (HR 3.61 (95% CI 2.61–4.99), *p* < 0.001) as independent risk factors for SARS-CoV-2 infection (Table 3). No independent association between any type of immunosuppressive agent and SARS-CoV-2 infection was observed.

**Table 3:** Multivariate analysis of SARS-CoV-2 infection risk.

	Odds ratio (95% CI)	*P*-value
Been within 1.5 m of confirmed Covid patient	3.61 (2.61; 4.99)	**< 0.001**
Medically treated diabetes	1.39 (1.05; 1.83)	**0.021**
Chronic lung disease	1.71 (1.13; 2.60)	**0.012**
Tacrolimus	0.98 (0.66; 1.44)	0.909
Tacrolimus off-peak level (µg/L)	0.98 (0.94; 1.02)	0.245
Cyclosporine	0.90 (0.59; 1.38)	0.638
Cyclosporine off-peak level (µg/L)	1.00 (1.00; 1.00)	0.566
Mycophenolate	0.81 (0.62; 1.07)	0.133
Mycophenolate daily dose (mg)	1.00 (1.00; 1.00)	0.488
Everolimus	1.12 (0.65; 1.91)	0.682
Everolimus peak-off level (µg/L)	1.03 (0.91; 1.17)	0.630
Corticosteroids (methylprednisone)	0.95 (0.68; 1.34)	0.779
Corticosteroids daily dose (mg)	0.99 (0.91; 1.07)	0.761

Results are obtained from a multivariate Cox proportional hazard model with missing data accounted by performing 10 multiple imputations.

Significant values are given in bold.

### Risk factors for severe COVID-19 in SOT recipients

92 (37.2%) out of 247 SARS-CoV-2 infected SOT recipients were admitted for hospitalization, of which 57 (23.1%) had severe COVID-19, defined as need for O_2_ supplementation, ICU admission or death. The COVID-19 related mortality rate was 7.3%. Of the 18 patients that died, 16 (88.9%) died in hospital. For the hospitalized patients in which the date of infection was know, 87.5% died within 30 days of SARS-CoV-2 infection infection (14/16).

Univariable logistic regression analysis showed (supplementary Table 2) that SOT recipients with severe COVID-19 were older (63.1 vs. 56.3, *p* = 0.024) and more often known with hypertension (94.7% vs. 71.89%, OR 5.95 (95% CI 1.91–18.52), *p* = 0.002), chronic kidney disease (66.7% vs. 38.0%, OR 3.20 (95% CI 1.72–5.95), p = < 0.001) and heart disease (33.3% vs. 15.26%, OR 2.76 (1.40–5.42), *p* = 0.003) compared to patients with non-severe COVID-19 (Table 4). Both baseline cyclosporin (19.3% vs. 7.58%, OR (95% CI 1.28–7.03), *p* = 0.012) and corticosteroid (68.42% vs. 44.0%, OR 2.72 (95% CI 1.46–5.09), *p* = 0.002) use was more frequent in the severe COVID-19 group. Severe COVID-19 patients more frequently had COVID-19 suspicion among housemates (38.07% vs. 11.74%, p = < 0.001) and close contact with a confirmed COVID-19 individual (40.0% vs. 22.4%, *p* = 0.010) than patients with non-severe COVID-19. Chronic kidney disease was not a risk factor for severe disease in the kidney transplant group (OR 2.23 (95% CI 0.71–5.88), *p* = 0.167), but was a risk factor in other SOT recipients (OR 2.90 (95% CI 1.43–5.880), *p* = 0.003).

**Table 4:** Characteristics of patients with SARS-CoV-2 infection versus severe COVID-19.

	SARS-CoV-2 infection	Severe COVID-19	*P*-value
Number of patients (n)	190	57	
Transplant			
Only kidney (%)	38 (20.21%)	20 (35.09%)	0.067
Only heart (%)	30 (15.84%)	9 (15.79%)	
Only liver (%)	60 (31.63%)	8 (14.04%)	
Only lung (%)	44 (23.16%)	16 (28.07%)	
Multi-organ (%)*	18 (9.16%)	4 (7.02%)	
Male (%)	111 (58.63%)	35 (61.40%)	0.733
Age (years)	56.3 (15.32)	63.1 (14.42)	**0.024**
BMI ≥ 30 (%)	34 (17.68%)	11 (19.30%)	0.746
Active or former smoker (%)	103 (54.26%)	37 (64.71%)	0.287
Hypertension (%)	137 (71.89%)	54 (94.74%)	**0.002**
Diabetes (%)	64 (33.89%)	23 (40.35%)	0.363
Chronic kidney disease (%)	72 (38.00%)	38 (66.67%)	**< 0.001**
Chronic heart disease (%)	29 (15.26%)	29 (33.33%)	**0.003**
Chronic lung disease (%)	20 (10.58%)	20 (14.04%)	0.431
Chronic liver disease (%)	6 (3.16%)	0 (0.00%)	0.378
Chronic neurological disease (%)	14 (7.47%)	4 (7.02%)	0.975
Current or past malignancy (solid organ or hematological; excl. skin tumors) (%)	10 (5.37%)	3 (5.26%)	0.996
Tacrolimus (%)	170 (89.26%)	46 (80.70%)	0.081
Tacrolimus off-peak level (µg/L; mean (SD))	5.7 (3.16)	5.6 (3.86)	0.357
Cyclosporine (%)	14 (7.58%)	11 (19.30%)	**0.012**
Cyclosporine off-peak level (µg/L; mean (SD))	9.4 (39.48)	18.9 (44.56)	**0.040**
Mycophenolate	106 (56.05%)	34 (59.65%)	0.597
Mycophenolate daily dose (mg; mean (SD))	555.2 (697.93)	606.0 (712.40)	0.597
Azathioprine (%)	20 (10.68%)	5 (8.77%)	0.700
Azathioprine daily dose (mg) (%)	6 (3.42%)	1 (1.75%)	0.902
Corticosteroids (methylprednisone) (%)	84 (44.00%)	39 (68.42%)	**0.002**
Corticosteroids daily dose (4 mg) (%)	54 (28.58%)	31 (54.39%)	**0.005**
Everolimus (%)	13 (6.95%)	3 (5.26%)	0.784
Everolimus peak-off level (µg/L; mean SD))	0.3 (1.17)	0.2 (0.67)	0.835
Number of housemates			
0 (%)	24 (12.72%)	5 (9.38%)	0.937
1 (%)	98 (51.83%)	30 (53.13%)	
> 1 (%)	67 (35.44%)	21 (37.50%)	
Covid suspicion among housemates (%)	22 (11.74%)	22 (38.07%)	**< 0.001**
Been within 1.5 m of confirmed COVID-199 case (%)	43 (22.37%)	23 (40.00%)	**0.010**
Always complied with COVID rules (%)	136 (71.48%)	33 (57.58%)	0.098
Housemates always complied with COVID rules (%)	103 (54.31%)	25 (43.75%)	0.359
Patient never bought food during complete lock-down (%)	85 (44.84%)	29 (50.88%)	0.472
Patient always wore mouth mask when social distance could not be kept (%)	163 (85.76%)	49 (85.29%)	0.825
Spent at least 1 night abroad since 4th February 2020 (%)	34 (18.11%)	10 (17.37%)	0.942

P-values are obtained from a logistic regression model with missing data accounted for by performing 10 multiple imputations.

*The multi-organ transplant group also contains 1 single-pancreas and 2 single intestine-patients.

Significant values are given in bold.

The median time from last transplant to onset of infection was 8.5 years. No significant relation with the severity of the disease could be detected (*p* = 0.275).

In multivariable logistic regression analysis, hypertension (OR 5.45 (95% CI 1.66–17.84), *p* = 0.005), chronic kidney disease (OR 3.55 (95% CI 1.75–7.19, *p* < 0.001), a housemate affected by COVID-19 (OR 6.77 (95% CI 2.65–17.28), *p* < 0.001) and baseline corticosteroid use (OR 2.93 (95% CI 1.03–2.55), *p* = 0.021) were identified as independent risk factors for severe COVID-19 (Table 5). Other immunosuppressive agents were not identified as an independent risk factor for severe COVID-19. No significant interaction (*p* = 0.91) between chronic kidney disease and transplant group could be shown (logistic regression analysis).

During the entire study period, most patients received corticosteroids (77%) and low-molecular weight heparin (64%) (if not on other anticoagulants) in case of COIVD-19 hospitalization. Remdezivir (14%) and hydroxychloroquine were less frequently administered (17%). If so, they were mostly administered during mid to late 2020. Further information regarding COVID-19 treatment can be found in the supplementary Table 3. Characteristics of COVID-19 related deaths can be found in supplementary Table 4.

**Table 5:** Multivariate analysis of severe vs. non-severe COVID-19.

	Comparison	Odds ratio (95% CI)	*P*-value
Transplant type			0.2813
	Only kidney vs. only liver	2.92 (1.05; 8.12)	**0.040**
	Only kidney vs. only lung	1.18 (0.47; 2.94)	0.723
	Only kidney vs. multi-organ	1.22 (0.32; 4.68)	0.767
	Only heart vs. only kidney	1.01 (0.35; 2.87)	0.988
	Only heart vs. only liver	2.94 (0.92; 9.46)	0.070
	Only heart vs. only lung	1.19 (0.43; 3.32)	0.741
	Only heart vs. multi-organ	1.23 (0.31; 4.89)	0.765
	Only liver vs. only lung	0.40 (0.14; 1.13)	0.084
	Only liver vs. multi-organ	0.42 (0.10; 1.78)	0.238
	Only lung vs. multi-organ	1.04 (0.28; 3.89)	0.956
Medically treated hypertension	Yes vs. no	5.45 (1.66;17.84)	**0.005**
Chronic kidney disease	Yes vs. no	3.55 (1.75; 7.19)	**< 0.001**
Housemates affected by COVID-19	Yes vs. no	6.77 (2.65;17.28)	**< 0.001**
Corticosteroids (methylprednisone)	Yes vs. no	2.55 (1.03 ;6.27)	**0.042**

Results are obtained from a logistic regression model with missing data by performing 10 multiple imputations.

*The multi-organ group also contains 1 single pancreas and 2 single intestine transplant recipients.

Significant values are given in bold.

## Discussion

In this large prospective cohort study in unvaccinated SOT recipients, no type of baseline immunosuppressive drug was identified as an independent risk factor for SARS-CoV-2 infection, in contrast to diabetes, chronic lung disease and close contact with a SARS-CoV-2 positive individual. However, baseline corticosteroid use was an independent risk factor for severe COVID-19 in SOT recipients, in addition to the traditional risk factors of hypertension, chronic kidney disease and close contact with a SARS-CoV-2 positive patient. Our data shed a new light on the multiple early studies, providing a more accurate insight in case-severity and case-mortality rates, and in the role of immunosuppressive agents regarding SARS-CoV-2 infection and severity in unvaccinated SOTs.

Intuitively, chronic immunosuppressive therapy may pose a theoretical increased risk for SARS-CoV-2, which would be in line with other viral infections^31^. Published data regarding risk factors for SARS-CoV-2 infection in unvaccinated SOT recipients are scarce. In line with studies in patients with IBD or rheumatic diseases, we did not find an association between immunosuppressive therapy and SARS-CoV-2 infection risk^32,33^. It is important to consider that patients receiving immunosuppressive therapy might have had better adherence to measures concerning SARS-CoV-2 infection, which theoretically could compensate their increased risk for acquiring a SARS-CoV-2 infection^34^. The observed adherence to government issued measures was overall high in our cohort and we did not find significant differences in adherence between infected and non-infected SOT recipients. The fact that COVID-19 suspicion among house mates and close contact with a confirmed COVID-19 individual were independent risk factors for infection and severe disease respectively, underlines the importance of preventive measures. Adherence to these measures is associated with lower SARS-CoV-2 infection rates, regardless of vaccination status^30^. Our data do not support temporarily discontinuing or altering immunosuppressive agents because of fear of acquiring SARS-CoV-2 infection. Both diabetes and chronic lung disease were independent risk factors for infection in our cohort, which is in line with results from the general population^35,36^, reflecting the validity of our analysis.

Due to our study design that included patients across the entire disease spectrum from asymptomatic to death, our data put the early morbidity and mortality data in perspective. Our hospitalization rates (37.2%), case severity (23.1%) and mortality (7.3%) of infected SOT recipients are considerably lower than what has often been reported in the literature stemming from the pre-vaccination era, with hospitalization and mortality rates as high as 78–89%^5,6^ and 18–30% respectively^7^.

Importantly, we identified baseline corticosteroid use as an independent risk factor for severe COVID-19 in SOT recipients, in addition to chronic kidney disease and hypertension. Given that corticosteroids are effective in reducing mortality during COVID-19^37^, this might seem contradictory. We hypothesize that the timing of corticosteroid use during SARS-CoV-2 infection is crucial and suspect that corticosteroids are disadvantageous during the initial replication phase of SARS-COV-2 enabling viral replication, but rather beneficial in the later phase of the infection which is marked by hyperinflammation. Given that both systemic and oral corticosteroids have been proposed to alter angiotensin converting enzyme-2 (ACE2) receptor expression^37–39^, this might be a possible explanation as to why specifically pre-infection corticosteroid use, as opposed to other immunosuppressive therapies, is associated with severe COVID-19. Additional studies are needed to draw more substantiated conclusions on this possible relationship. Similar to our findings, registry studies in IBD and rheumatological patients also identified corticosteroid use as a risk factor for severe COVID-19^38,40^ in unvaccinated patients, although at higher dosages (> 10 mg of prednisone) than the mean 4 mg of methylprednisone a day in our SOT recipients. The risk of corticosteroid use is most clinically relevant for lung and kidney transplant recipients who most frequently received corticosteroids (99.3% and 58.3% respectively). In line, a retrospective cohort of unvaccinated SOTs (*n* = 600), both lung and kidney transplant recipients were at higher risk of severe COVID-19 than liver transplant recipients^41^.

Apart from corticosteroids, we did not identify tacrolimus, MMF or other immunosuppressive regiments as either protective or disadvantageous for severe COVID-19 in unvaccinated transplant recipients. This result is in contrast with earlier studies reporting protective effects for tacrolimus^23^ and disadvantageous effects for MMF^24^, but is in line with others^22,25,26^.

The major limitation of our analysis is that the study was performed in the beginning of the pandemic where Alpha, Beta and Gamma variants were dominant^42^ and that we did not include immunocompetent controls. On the other hand, it is a strength that we assessed a very large cohort of never-vaccinated SOTs, which is a unique study context which will not be possible to recreate to that extent in the near future. A further strength of our analysis is the large study population, including all types of SOTs. We used both RT-PCR and SARS-CoV-2 IgG antibody testing for the assessment of the SARS-CoV-2 infection enabling the inclusion of patients across the entire disease spectrum and enlarging the diagnostic time window after infection. The consecutive inclusion of SOT recipients enabled us to form a large control group of non-infected individuals in contrast to registry studies with solely infected SOT recipients. We are aware of only one case of COVID-19 related death that was not included in our study, limiting the risk of immortal time bias. However, some SARS-CoV-2 infections may have been missed in case of waning of IgG antibodies. Our data are relevant for unvaccinated SOT recipients, SOT recipients not willing to undergo repetitive vaccine doses and SOT recipients with lack of vaccine immune response.

In conclusion, we found no significant association between baseline immunosuppressive therapy and SARS-CoV-2 infection risk in unvaccinated SOT recipients, whereas only baseline corticosteroid use is independently associated with severe COVID-19 in these patients. Based on these study data in unvaccinated SOTs and our previously published study in vaccinated SOTs^15^, which were performed in the same cohort, we are able to provide an integral overview on the role of different types of immunosuppressive agents and other risk factors in SARS-CoV-2 infection and vaccine response in Table 6^15^. Taken together, no significant association between immunosuppressive baseline therapy and SARS-CoV-2 infection in unvaccinated patients was found, but corticosteroids are a risk factor for SARS-CoV-2 infection in vaccinated SOTs. Furthermore, baseline corticosteroid use is associated with severe COVID-19 regardless of vaccination status. MMF use is only a risk factor for severe COVID-19 in vaccinated SOTs. Lastly, both corticosteroid and MMF use are related to diminished humoral vaccine response in SOTs^15^. These data suggest that corticosteroids are a risk factor for severe COVID-19 due to both their negative impact on vaccine response and via more direct pro-viral immunological effects.

**Table 6:** Summary of effects of immunosuppressive therapy and other risk factors on SARS-CoV-2 infection and severe COVID-19 in vaccinated and unvaccinated patients^15^.

Independent risk factor yes/no	SARS-CoV-2 infection (pre-vaccination)	Severe COVID-19 (pre-vaccination)	No humoral vaccine response (after 3rd dose)	SARS-CoV-2 Infection (post-vaccination)	Severe COVID-19 (post-vaccination)
Corticosteroids (methylprednisone)	No	Yes	Yes	Yes	Yes
Mycophenolate	No	No	Yes	No	Yes
Tacrolimus	No	No	No	No	No
Cyclosporin	No	No	No	No	No
Everolimus	No	No	No	No	No
Azathioprine	No	No	No	No	No
Other risk factors	Close contact with COVID-19 patient, diabetes and lung disease	Housemate with confirmed COVID-19, chronic kidney disease, hypertension	Older age, chronic kidney disease	Younger age, shorter interval to transplantation, and only 1 vaccination dose	Older age

## Methods

### Patient inclusion, variables and definitions

The first SARS-CoV-2 case in Belgium was reported on the 4th of Febuary marking the start of the COVID-19 pandemic in Belgium^43^. In this prospective study, SOT recipients were consecutively included between July 3, 2020 and April 14, 2021 during their outpatient visit to their transplant physician in the context of standard medical care. Follow-up visits were performed between August 24, 2020 and May 10th, 2021. In March 2020, the first lockdown took place with a strict curfew and closure of all non-essential facilities^44^. The first SARS-CoV-2 PCR tests were performed in a selected population (hospitalized patients/specific workers with suspect symptoms). In July 2020 during the start of the study period, the first lockdown was lifted and (limited) cross-country travel was allowed. From August 2020 facemasks were required to be worn in all public places. In September 2020 this was reduced to wearing masks only on public transport, in shops and cinemas. Furthermore, testing was recommended for anybody with symptoms. In October 2020, a second lockdown (closure of non-essential facilities) was instilled after a rise in SARS-COV-2 incidence and remained until April 2021 ^44^. Furthermore, traveling was restricted until April 2021, with required testing and quarantining. Self-testing only became common practice at the end of 2021, although the first test kits were available from April 2021. Social distancing rules were in place during the entire study period.

Follow-up visits were performed between August 24, 2020 and May 10th 2021. All adult patients (age > 18 year) who underwent SOT (including liver, kidney, lung, heart or combined transplantation) were eligible. Patients were followed-up at 3, 6 and 12 months.

Vaccination status was checked using the Belgian “vaccinet” database and self-reported by patients. Patients were censored at time of vaccination or excluded. Although the first SARS-CoV-2 vaccinations were performed in January 2021 (for nursing home residents)^44^, most transplant recipients received their first vaccination at the end of April or beginning of May 2021. All methods were carried out in accordance with relevant guidelines and regulations. The study protocol was approved by the UZ Leuven ethical commission.

Informed consent was obtained from all subjects, after which a questionnaire regarding diagnosis of prior SARS-CoV-2 infection and risk factors regarding adherence to COVID-19 rules was collected. SARS-CoV-2 anti-nucleocapsid (N) IgG were measured in all patients at the time of inclusion. In 1403/1957 (71.7%) patients we also measured anti-spike (anti-S) IgG antibodies. The diagnosis of SARS-CoV-2 infection was defined as a positive real time polymerase chain reaction (RT-PCR) test via nasopharyngeal swab and/or bronchoalveolar lavage (BAL) and/or a positive SARS-CoV-2 anti-spike (anti-S) IgG antibody and/or positive SARS-CoV-2 anti-nucleocapsid (anti-N) IgG antibody in the past or at the moment of inclusion. The absence of SARS-CoV-2 infection was defined as a negative SARS-CoV-2 anti-S IgG antibody titer, negative SARS-CoV-2 anti-N IgG antibody titer (both in the past and at the moment of inclusion) and the absence of a positive RT-PCR via nasopharyngeal swab or BAL. The results of SARS-CoV-2 RT-PCR or antibody analyses (if performed) before study inclusion were all cross-checked via the electronic medical file of the patient. Detailed information regarding type, dose and trough levels of immunosuppressive therapy were collected within standard medical care. In case immunosuppressive therapy was adapted during follow up (for other reasons than COVID-19), an average dose over the study period was used for analysis. Comorbidities were collected from the electronic medical file and defined as the following: arterial hypertension (receiving antihypertensive drugs), diabetes mellitus (receiving anti-diabetic drugs), chronic kidney disease (defined as an Cockcroft-Gault eGFR of < 60 ml/min during > 6 months), chronic heart disease (myocardial infarction, congestive heart failure or transplant vasculopathy), chronic lung disease (asthma, chronic obstructive pulmonary disease, emphysema, sarcoidosis, interstitial lung disease or chronic lung allograft dysfunction, chronic liver disease (cirrhosis), neurological disease (history of cerebrovascular accident, transient ischemic attack, dementia, Parkinson’s disease or multiple sclerosis), human immunodeficiency virus (HIV) infection and finally solid and/or hematological malignancies in the last 5 years (excluding non-metastatic basal and squamous carcinomas of the skin, including pre-transplantation malignancies such as hepatocellular carcinoma).

Severe COVID-19 was defined as the need for in-hospital oxygen (O_2_) supplementation (room air oxygen saturation < 90%), intensive care unit admission (ICU) or COVID-19 related death^45^. In case of hospitalization, data on the length of stay, need for supplemental oxygen, non-invasive ventilation (defined as continuous positive airway pressure / bi-level positive airway pressure and/or high flow nasal cannula), admission to the intensive care unit along with length of stay, invasive ventilation, need for vasopressor therapy and need for renal replacement therapy were collected. In case hospital admission was before the first baseline visit, data regarding hospitalization were retrospectively collected. Additionally, targeted treatment for COVID-19, including administration of low weight molecular heparin (LWMH), and preventive in-hospital modulation of immunosuppressive agents because of COVID-19 were collected (Supplementary Table 1). Moreover, data regarding organ rejection and treatment for organ rejection after admission for COVID-19 were collected.

The study was conducted at the University Hospitals of Leuven and a limited number of liver (*n* = 9) and kidney (*n* = 69) transplant recipients were included at the affiliated hospitals of ZOL Genk and Jessa Hospital Hasselt, respectively (all these patients underwent organ transplantation at UZ Leuven). The study was approved by the Ethical Committee of University Hospitals Leuven (S64036) and is registered on Clinicaltrials.gov (NCT04579471). The study was conducted in accordance with the ethical principle’s declaration of Helsinki.

### Antibody assays

SARS-CoV-2 anti-N and anti-S IgG analyses at the time-point of patient inclusion were performed using Abbot’s SARS-CoV-2 IgG and IgG II Quant chemiluminescent immunoassays (CLIA) on the Architect I2000SR (Abbot, USA). In line with the manufacturer’s recommendations, an anti-N IgG signal / cut off (S/CO) value of ≥ 1.40 was considered positive. For anti-S IgG, a value of ≥ 50 arbitrary units per ml (AU/ml) was considered positive. Additionally, EUROIMMUN Anti-SARS-CoV-2 QuantiVac ELISA on the EUROIMMUN Analyzer 1 (Luebeck, Germany) was used. Values above 10 relative units per millilitre (RU/ml) were considered positive. In case of discrepancy between the EUROIMMUN and Abbot SARS-CoV-2 anti-S IgG positivity, samples were retested (*n* = 40). There were no discrepancies after the retesting.

### Statistical analysis

Baseline characteristics are described descriptively overall, and by transplant type using the mean and standard deviation or median and interquartile range for continuous variables. For categorical variables numbers and percentages were used. Multiple imputation using 10 imputations was performed to impute missing data for the baseline characteristics, the follow-up visits data and blood tests at the different visits. Only for performed visits, data were imputed. The fully conditional specification using the logistic regression and discriminant function method for categorical variables and the regression method for continuous variables was applied. For Cox regression analysis, time zero was 4 February 2020 (first case of COVID-19 in Belgium) or date of last transplantation before the baseline visit, wfehichever comes last. Patients who had a SARS-CoV-2 infection before transplantation were excluded from the study. If the date of infection was not available, data was considered to be interval censored between the last available visit without infection or time 0, and the visit date at which a prior SARS-CoV-2 infection was confirmed. The interval censoring was dealt with by multiple times imputing a random event time between the lower and upper limit of the event. For most covariates, the questionnaire information obtained at the first visit was used as baseline variables. For immunosuppressive therapy, the average dose (including changes since February 4th 2020) up to SARS-COV-2 infection or censoring was calculated from all available visits and used as a baseline value. No specific competing risk methodology was applied to account for death or re-transplantation due to the low number of deaths (*n* = 18). There was no case of re-transplantation during the study period.

Risk factors for SARS-CoV-2 infection were investigated using a Cox proportional hazards model. The proportional hazards (PH) assumption was verified using the graphical methods of Lin, Wei, and Ying (1993) (ASSESS statement in PROC PHREG). For some transplant types, the PH assumption was violated. However, the PH assumption was met for the other variables. A sensitivity analysis using a model stratifying on transplant type yielded similar estimates for the other variables. We opted to report the model with transplant group as a covariate in order to provide hazard ratios for the different transplant types. Patients without a SARS-CoV-2 infection were censored at the last available visit. At the time of analysis, COVID-19 was a new spreading disease for which clear literature guidance was limited, making pre-specification difficult. Therefore all covariates that univariably had a p-value < 0.2, were investigated in a multivariable model. A p-value < 0.05 was used to stay in the model. For univariable analyses, no adjustment was performed. For multivariable analysis regarding SARS-CoV-2 infection, we finally adjusted for: transplant type, hypertension, chronic lung disease, COVID-19 suspicion among housemates and been with < 1.5 m of confirmed COVID-19 case.

Second order interactions and linearity assumption of continuous covariates were verified. The hazard ratios (HR) with a 95% confidence interval (CI) were reported.

Among all patients that had a SARS-CoV-2 infection; risk factors for severe COVID-19 were investigated using a logistic regression model. Severe COVID-19 was defined as O_2_ need during a hospitalization and/or ICU admission and/or death from COVID-19. All covariates that univariably had a p-value < 0.2, were investigated in a multivariable model. Transplant type was forced into the model. Backward selection was applied to get a parsimonious model. A p-value < 0.05 was used to stay in the model. For multivariable analysis we finally adjusted for: transplant type, hypertension, chronic kidney disease, corticosteroid use and dose, COVID-19 suspicion among housemates and if patients was within < 1,5 m of confirmed COVID-19 case. Second order interactions and linearity assumption of continuous covariates were verified. The odds ratios (OR) with a 95% CI were reported. All analyses were performed using SAS/STAT software version 9.4 for Windows (SAS institute Inc., USA).

## Electronic supplementary material

Below is the link to the electronic supplementary material.

Supplementary Material 1

## Data Availability

Data used in this study can be assesed upon reasonable request to the corresponding author.

## Abbreviations

ACE2: Angiotensin converting enzyme-2
Anti-N: Anti-nucleocapsid
Anti-S: Anti-spike
BAL: Bronchoalveolar lavage
CNI: Calcineurin inhibitors (CNI)
ICU: Intensive care unit
IBD: Inflammatory bowel disease
LMWH: Low weight molecular heparin
MMF: Mycophenolate mofetil
O_2_: In-hospital oxygen
SARS-CoV-2: Severe acute respiratory syndrome coronavirus 2
SOTs: Solid organ transplant recipients
