# Supplementary material for: Risk factors for SARS-CoV-2 infection and severe COVID-19 in unvaccinated solid organ transplant recipients
Source: Sci Rep. 2024 Nov 2;14:26465. doi: 10.1038/s41598-024-78119-6 (PMC11531510; doi:10.1038/s41598-024-78119-6)
Supplement: Supplementary file 1 — Supplementary Material 1 [file 41598_2024_78119_MOESM1_ESM.docx]

**Supplementary table 1: Univariable analysis of SARS-CoV-2 infection risk (Cox proportional hazard model).**

|  | **Hazard Ratio (95% CI)** | **P-value** |
| --- | --- | --- |
| Type of transplant |  |  |
| Only kidney vs Only liver | 0.75 (0.52; 1.09) | 0.127 |
| Only kidney vs Only lung | 0.82 (0.57; 1.19) | 0.307 |
| Only kidney vs Multi-organ | 0.82 (0.48; 1.40) | 0.470 |
| Only heart vs Only kidney | 1.45 (0.96; 2.21) | 0.080 |
| Only heart vs Only liver | 1.09 (0.72; 1.65) | 0.683 |
| Only heart vs Only lung | 1.20 (0.80; 1.80) | 0.387 |
| Only heart vs Multi-organ | 1.20 (0.69; 2.08) | 0.525 |
| Only liver vs Only lung | 1.10 (0.76; 1.59) | 0.617 |
| Only liver vs Multi-organ | 1.10 (0.64; 1.87) | 0.732 |
| Only lung vs Multi-organ | 1.00 (0.59; 1.69) | 0.995 |
| Gender | 0.96 (0.75; 1.25) | 0.781 |
| BMI >=30 | 1.06 (0.75; 1.50) | 0.754 |
| Active or former smoker | 0.93 (0.69; 1.26) | 0.633 |
| Hypertension | 0.72 (0.53; 0.98) | 0.038 |
| Diabetes | 1.40 (1.07; 1.82) | 0.013 |
| Chronic kidney disease | 0.84 (0.65; 1.09) | 0.194 |
| Chronic heart disease | 1.16 (0.85; 1.60) | 0.347 |
| Chronic lung disease | 1.96 (1.32; 2.92) | <.001 |
| Chronic liver disease | 1.96 (0.86; 4.42) | 0.107 |
| Chronic neurological disease | 1.16 (0.71; 1.87) | 0.556 |
| Current or past malignancy (solid organ or hematological). | 0.71 (0.40; 1.25) | 0.230 |
| Chemo-/immunotherapy against malignancy in 2020 | 0.00 (0.00;7,266) | 0.972 |
| Tacrolimus | 1.08 (0.74; 1.58) | 0.696 |
| Cyclosporine | 0.83 (0.54; 1.26) | 0.380 |
| Cyclosporine off-peak level (µg/L) | 1.00 (0.99; 1.00) | 0.387 |
| Mycophenolate mofetil | 0.77 (0.60; 1.01) | 0.055 |
| Azathioprine | 0.93 (0.61; 1.41) | 0.727 |
| Corticosteroids daily dose. | 0.91 (0.68; 1.21) | 0.518 |
| Everolimus | 1.10 (0.65; 1.85) | 0.724 |
| Everolimus peak-off level (µg/L) | 1.04 (0.91; 1.17) | 0.572 |
| Number of housemates. | 1.03 (0.67; 1.58) | 0.898 |
| COVID-19 suspicion among housemates | 3.30 (2.28; 4.78) | <.001 |
| Been within 1.5m of confirmed COVID-19 case. | 3.71 (2.72; 5.07) | <.001 |
| Always complied with COVID rules | 0.91 (0.68; 1.23) | 0.551 |
| Housemates always complied with COVID rules | 1.05 (0.78; 1.41) | 0.750 |
| Patient never bought food during complete lock-down | 1.29 (0.99; 1.68) | 0.057 |
| Patient always wore mouth mask when social distance could not be kept. | 0.77 (0.52; 1.13) | 0.181 |

**Supplementary table 2: Univariable analysis for severe COVID-19 (Logistic regression model).**

|  | **Odds Ratio (95% CI)** | **P-value** |
| --- | --- | --- |
| Transplant |  |  |
| Only heart vs Only kidney | 0.58 (0.23; 1.46) | 0.250 |
| Only heart vs Only liver | 2.22 (0.79; 6.23) | 0.131 |
| Only heart vs Only lung | 0.84 (0.33; 2.13) | 0.714 |
| Only heart vs Multi-organ | 1.21 (0.33; 4.41) | 0.771 |
| Only kidney vs Only liver | 3.79 (1.54; 9.35) | 0.004 |
| Only kidney vs Only lung | 1.44 (0.65; 3.15) | 0.366 |
| Only kidney vs Multi-organ | 2.07 (0.63; 6.81) | 0.231 |
| Only liver vs Only lung | 0.38 (0.15; 0.95) | 0.039 |
| Only liver vs Multi-organ | 0.55 (0.15; 1.97) | 0.356 |
| Only lung vs Multi-organ | 1.44 (0.43; 4.80) | 0.551 |
| Gender | 1.11 (0.61; 2.03) | 0.733 |
| BMI >=30 | 1.14 (0.52; 2.49) | 0.746 |
| Active or former smoker | 1.52 (0.71; 3.26) | 0.287 |
| Hypertension | 5.95 (1.91;18.52) | 0.002 |
| Diabetes | 1.33 (0.72; 2.43) | 0.363 |
| Chronic kidney disease | 3.20 (1.72; 5.95) | <.001 |
| Chronic heart disease | 2.76 (1.40; 5.42) | 0.003 |
| Chronic lung disease | 1.42 (0.59; 3.40) | 0.431 |
| Chronic liver disease | 0.25 (0.01; 5.56) | 0.378 |
| Chronic neurological disease | 1.02 (0.33; 3.15) | 0.975 |
| Current or past malignancy (solid organ or hematological; excl. skin tumors). | 1.00 (0.28; 3.57) | 0.996 |
| Tacrolimus | 0.49 (0.22; 1.09) | 0.081 |
| Cyclosporine | 2.99 (1.28; 7.03) | 0.012 |
| Mycophenolate | 1.18 (0.65; 2.14) | 0.597 |
| Azathioprine | 0.82 (0.30; 2.24) | 0.700 |
| Corticosteroids | 2.72 (1.46; 5.09) | 0.002 |
| Corticosteroids daily dose (4mg) | 3.33 (1.71; 6.46) | <.001 |
| Everolimus | 0.84 (0.24; 2.93) | 0.784 |
| Number of housmates | 0.97 (0.43; 2.16) | 0.937 |
| Covid suspicion among housemates | 4.86 (2.25;10.50) | <.001 |
| Been within 1.5m of confirmed Covid patient | 2.31 (1.22; 4.36) | 0.010 |
| Always compliance with COVID rules | 0.53 (0.25; 1.13) | 0.098 |
| Always compliance of housemates (+16y) with COVID rules | 1.74 (0.79; 3.86) | 0.171 |
| Patient bought never food during complete lock-down | 1.27 (0.66; 2.45) | 0.472 |
| Patient wears always mouth mask if social distance of 1.5m cannot be kept. | 0.89 (0.32; 2.47) | 0.825 |

**Supplementary table 3: Characteristics of hospitalized patients, immunosuppressive changes and organ rejection during and after confirmed SARS-CoV-2 infection.**

| **Transplant type** | | | | | | |  |
| --- | --- | --- | --- | --- | --- | --- | --- |
|  | **Only kidney** | **Only heart** | **Only liver** | **Only lung** | **Multi-organ** | **Total** |  |
| Hospitalization due to COVID-19 | 27/566 (4.8%) | 15/318 (4.7%) | 12/326 (3.7%) | 28/566 (4.9%) | 7/178 (3.9%) | 92/1967 (4.7%) |  |
| Duration of hospitalization (days) – Median (Q1 – Q3) | 9 (5 ; 27) | 6 (5 ; 11) | 10 (5 ; 19) | 8 (5 ; 18) | 8 (8 ; 16) | 8 (5 ; 18) |  |
| Need for O_2_ during hospitalization | 18/25 (72.0%) | 9/15 (60.0%) | 8/12 (66.7%) | 15/26 (57.7%) | 3/7 (42.9%) | 54/88 (61.4%) |  |
| Non-invasive mechanical ventilation (CPAP/BiPAP) | 4/25 (16.0%) | 3/15 (20.0%) | 4/12 (33.3%) | 5/26 (19.2%) | 2/7 (28.6%) | 20/88 (22.7%) |  |
| Vasopressin | 3/25 (12.0%) | 0/15 (0.0%) | 1/12 (8.3%) | 3/26 (11.5%) | 2/7 (28.6%) | 10/88 (11.4%) |  |
| Dialysis | 2/25 (8.0%) | 0/15 (0.0%) | 2/12 (16.7%) | 1/26 (3.8%) | 3/7 (42.9%) | 8/88 (9.1%) |  |
| Intensive care | 7/25 (28.0%) | 0/15 (0.0%) | 2/12 (16.7%) | 5/26 (19.2%) | 3/7 (42.9%) | 19/88 (21.6%) |  |
| Duration of ICU stay (days) – Median (Q1 – Q3) | 19 (5 ; 24) | - | 19 (18 ; 20) | 14 (13 ; 27) | 13 (1 ; 14) | 14 (7 ; 21) |  |
| **COVID-19 treatment during hospitalisation** | 25/564 (4.4%) | 17/318 (5.3%) | 12/326 (3.7%) | 27/563 (4.8%) | 8/178 (4.5%) | 92/1962 (4.7%) |  |
| Hydroxychloroquine | 5/25 (20.0%) | 0/16 (0.0%) | 0/12 (0.0%) | 7/27 (25.9%) | 1/8 (12.5%) | 15/91 (16.5%) |  |
| Azithromycine | 1/25 (4.0%) | 0/16 (0.0%) | 2/12 (16.7%) | 25/27 (92.6%) | 4/8 (50.0%) | 33/91 (36.3%) |  |
| Remdesevir | 2/25 (8.0%) | 0/16 (0.0%) | 0/12 (0.0%) | 9/27 (33.3%) | 2/8 (25.0%) | 13/91 (14.3%) |  |
| Corticosteroids | 18/25 (72.0%) | 10/17 (58.8%) | 7/12 (58.3%) | 27/27 (100.0%) | 7/8 (87.5%) | 71/92 (77.2%) |  |
| LMWH | 14/25 (56.0%) | 11/17 (64.7%) | 10/12 (83.3%) | 17/27 (63.0%) | 4/8 (50.0%) | 59/92 (64.1%) |  |
| **Change in immunosupressive therapy dosage during SARS-CoV-2 infection (compared to baseline)** | 18/25 (72.0%) | 5/17 (29.4%) | 7/12 (58.3%) | 18/27 (66.7%) | 6/8 (75.0%) | 55/90* (61.1%) |  |
| **Tacrolimus** | | | | | |  |  |
| Increased | 0/0 (0.0%) | 1/1 (100.0%) | 2/4 (50.0%) | 1/3 (33.3%) | 0/1 (0.0%) | 4/9 (44.4%) |  |
| Decreased | 0/0 (0.0%) | 0/1 (0.0%) | 2/4 (50.0%) | 2/3 (66.7%) | 1/1 (100.0%) | 5/9 (55.6%) |  |
| **Cyclosporines** | | | |  |  |  |  |
| Increased | 0/1 (0.0%) | 0/0 (0.0%) | 0/0 (0.0%) | 0/1 (0.0%) | 0/0 (0.0%) | 0/2 (0.0%) |  |
| Decreased | 1/1 (100.0%) | 0/0 (0.0%) | 0/0 (0.0%) | 1/1 (100.0%) | 0/0 (0.0%) | 2/2 (100.0%) |  |
| **Mycophenolate Mofetil** | | |  |  |  |  |  |
| Increased | 1/14 (7.1%) | 0/4 (0.0%) | 0/5 (0.0%) | 0/10 (0.0%) | 0/4 (0.0%) | 1/38 (2.6%) |  |
| Decreased | 13/14 (92.9%) | 4/4 (100.0%) | 5/5 (100.0%) | 10/10 (100.0%) | 4/4 (100.0%) | 37/38 (97.4%) |  |
| **Azathioprine** | | |  |  |  |  |  |
| Increased | 0/1 (0.0%) | 0/0 (0.0%) | 0/0 (0.0%) | 0/4 (0.0%) | 0/1 (0.0%) | 0/6 (0.0%) |  |
| Decreased | 1/1 (100.0%) | 0/0 (0.0%) | 0/0 (0.0%) | 4/4 (100.0%) | 1/1 (100.0%) | 6/6 (100.0%) |  |
| **Everolimus** | | |  |  |  |  |  |
| Increased | 0/0 (0.0%) | 0/0 (0.0%) | 0/1 (0.0%) | 0/1 (0.0%) | 0/0 (0.0%) | 0/2 (0.0%) |  |
| Decreased | 0/0 (0.0%) | 0/0 (0.0%) | 1/1 (100.0%) | 1/1 (100.0%) | 0/0 (0.0%) | 2/2 (100.0%) |  |
| **Corticosteroids** | | |  |  |  |  |  |
| Increased | 6/6 (100.0%) | 1/1 (100.0%) | 1/1 (100.0%) | 5/5 (100.0%) | 2/2 (100.0%) | 15/15 (100.0%) |  |
| Decreased | 0/6 (0.0%) | 0/1 (0.0%) | 0/1 (0.0%) | 0/5 (0.0%) | 0/2 (0.0%) | 0/15 (0.0%) |  |
| **Organ rejection during PCR confirmed SARS-COV-2 infection** | 3/37 (8.1%) | 4/28 (14.3%) | 0/42 (0.0%) | 11/47 (23.4%) | 3/11 (27.3%) | 21/171 (12.3%) |  |
| Acute rejection | 1/3 (33.3%) | 0/4 (0.0%) | 0/0 (0.0%) | 2/11 (18.2%) | 0/3 (0.0%) | 3/21 (14.3%) |  |
| Cellular rejection | 1/1 (100.0%) | 0/0 (0.0%) | 0/0 (0.0%) | 1/2 (50.0%) | 0/0 (0.0%) | 2/3 (66.7%) |  |
| Antibody mediated rejection | 0/1 (0.0%) | 0/0 (0.0%) | 0/0 (0.0%) | 1/2 (50.0%) | 0/0 (0.0%) | 1/3 (33.3%) |  |
| Chronic rejection | 2/3 (66.7%) | 4/4 (100.0%) | 0/0 (0.0%) | 9/11 (81.8%) | 3/3 (100.0%) | 18/21 (85.7%) |  |
| **Organ rejection after PCR confirmed SARS-CoV-2 infection** | 4/36 (11.1%) | 2/26 (7.7%) | 0/42 (0.0%) | 9/45 (20.0%) | 3/11 (27.3%) | 18/166 (10.8%) |  |
| Acute rejection | 2/4 (50.0%) | 0/2 (0.0%) | 0/0 (0.0%) | 2/10 (20.0%) | 0/3 (0.0%) | 4/19 (21.1%) |  |
| Cellular rejection | 2/2 (100.0%) | 0/0 (0.0%) | 0/0 (0.0%) | 1/3 (33.3%) | 0/0 (0.0%) | 3/5 (60.0%) |  |
| Antibody mediated rejection | 0/2 (0.0%) | 0/0 (0.0%) | 0/0 (0.0%) | 2/3 (66.7%) | 0/0 (0.0%) | 2/5 (40.0%) |  |
| Chronic rejection | 2/4 (50.0%) | 2/2 (100.0%) | 0/0 (0.0%) | 8/10 (80.0%) | 3/3 (100.0%) | 15/19 (78.9%) |  |

*in 2 cases no information regarding immunosuppressive therapy during hospitalization was available.

**Supplementary table 4: Characteristics of patients with COVID-19 related death**

|  |  |
| --- | --- |
| **Characteristics** | **COVID related death** |
| Male (n/N (%)) | 13/18 (72.2%) |
| Age (years) median | 74 (65; 80) |
| Obesity (BMI>=30 kg/m²) | 1/16 (6.3%) |
| Current smoking |  |
| Active | 0/6 (0.0%) |
| Former | 4/6 (66.7%) |
| Never | 2/6 (33.3%) |
| Total n. of transplants |  |
| 1 | 14/18 (77.8%) |
| 2 | 2/18 (11.1%) |
| 3 | 0/18 (0.0%) |
| 4 | 2/18 (11.1%) |
| 5 | 0/18 (0.0%) |
